# Examining the dimensionality of pre-service teachers’ enthusiasm for teaching by combining frameworks of educational science and organizational psychology

**DOI:** 10.1371/journal.pone.0259888

**Published:** 2021-11-18

**Authors:** Denise Bock, Ute Harms, Daniela Mahler

**Affiliations:** 1 Biology Education, IPN—Leibniz Institute for Science and Mathematics Education, Kiel, Germany; 2 Biology Education, Freie Universität Berlin, Berlin, Germany; National Taiwan University of Science and Technology, TAIWAN

## Abstract

The aim of this study was to obtain a holistic understanding of pre-service teachers’ enthusiasm for teaching (a subject) by examining its structure as well as relevant factors that may be related to it in the first phase of teacher education. For this purpose, we considered two strands of research: educational science and organizational psychology. Accordingly, the professional competence model and the job demands and resources model helped to identify factors that are associated with pre-service teachers’ enthusiasm for teaching. Responses of 211 pre-service biology teachers indicated that enthusiasm for teaching can be considered as one-dimensional. Moreover, we found positive relationships between enthusiasm for teaching and academic self-concept, intrinsic career choice motives and occupational commitment. In contrast, we detected negative relations between enthusiasm for teaching and both emotional exhaustion and intention to quit. No significant relations could be found for enthusiasm for teaching and both professional knowledge and extrinsic career choice motives. Our findings highlight the importance of enthusiasm for teaching in the earliest stage of teachers’ careers. Thus, our study points out relevant factors that could help to maintain high enthusiasm and to keep (pre-service) teachers healthy and in the profession.

## Introduction

Descriptions of ‘ideal teachers’ frequently include *competence*. However, the most competent teachers only assist students’ learning if they are healthy and stay in the profession. High rates of teachers’ absenteeism and attrition also suggest that teachers’ health and well-being are important [1–4] and should be fostered as early as possible. Moreover, *enthusiasm of teachers* is a key element of their competence and health, for several reasons. First, as an affective motivational orientation, enthusiasm is an important component of teachers’ professional competence [5–7]. Teachers’ enthusiasm enhances students’ enjoyment, interest and motivation [8–12]. It is also positively related to instructional quality [13, 14], which strongly influences students’ performance [15, 16], and desirable student-teacher relationships [6, 9, 17].

Second, enthusiasm is an important health**-**promoting personal resource for teachers [18, 19]. Overwork and burnout impair not only teachers’ health but also their performance, and hence instructional quality, students’ performance and motivation, and schools at organizational levels [20–22]. In contrast, high enthusiasm potentially counteracts these effects [20, 23].

Recent studies have treated enthusiasm either as an element of professional competence [e.g. 6, 7, 16] or a personal resource that promotes teachers’ health [17, 24, 25]. Furthermore, little is known about the enthusiasm of pre-service teachers. However, key foundations of teachers’ competence, like professional knowledge and affective-motivational orientations, are established during the academic phase of teacher education [5, 26]. Additionally, teachers’ early career is formative as it is very demanding and related to emotional exhaustion [3, 27–29]. To explore pre-service teachers’ enthusiasm for teaching holistically, this study identifies factors that promote and interact with enthusiasm for teaching. We refer to two models derived from research on educational science and organizational psychology, respectively: the teachers’ professional competence model (TPC model; [5]) and the job demands and resources model (JD-R model; [30]). The TPC model concerns characteristics required for successful teaching (e.g., professional knowledge, enthusiasm), while the JD-R model is frequently used to assess factors and processes related to teachers’ burnout and work engagement, and is also including enthusiasm [18, 23, 31]. By considering these different perspectives, we aim to illuminate and link key factors more strongly than with a single point of view.

To be able to examine enthusiasm-related factors, insight on the conceptualization and dimensionality of teacher enthusiasm is needed. Research has shown that teacher enthusiasm comprises enthusiasm for the subject and enthusiasm for teaching [6, 7, 32]. However, there is an inconsistent and unclear use of terminology concerning the latter, as it is described either as “enthusiasm for teaching” and/or “enthusiasm for teaching the subject” [6, 13, 33]. Thus, we first ask the question whether enthusiasm for teaching and enthusiasm for teaching the subject are two different terms for the same construct, or whether they can be used to describe two distinguishable constructs.

### Enthusiasm for teaching

#### Conceptualization

Beside the long tradition of considering teacher enthusiasm in terms of instructional behavior, which influences student’s motivation and performance [8, 12, 25, 34–37], a second conceptualization considers teacher’s self-reported enthusiasm as a factor of a positive affective orientation manifested in teachers’ joy and excitement about their subject and teaching [6, 7, 32]. Keller and colleagues [32] even propose use of the terms ‘enjoyment’ or ‘passion’ rather than ‘enthusiasm’. However, as enthusiasm is conceptually closely related to intrinsic motivation, it has–unlike an emotion such as ‘joy’–an action-related component [7], thus, we prefer the term *enthusiasm*. As teachers’ internal processes are particularly important for their health (see the JD-R model; [18, 23, 30, 38, 39]), we apply the second conceptualization of enthusiasm, based on pre-service teachers self-reported experience.

#### Dimensionality

Before attempting to identify determinants of enthusiasm for teaching or its interaction with other factors, it is important to consider its dimensionality. Various studies that have adopted an affective conceptualization distinguish two dimensions: content-related *enthusiasm for the subject* and activity-related *enthusiasm for teaching* [6, 7, 16, 24, 40]. While there is consensus concerning the definition of *enthusiasm for the subject* as a “topic-related affective orientation” [33], the conceptualization and operationalization of *enthusiasm for teaching* are inconsistent. Kunter and colleagues [6] define it both as enthusiasm for teaching *per se* and enthusiasm for teaching a specific subject. Aldrup and colleagues [24] measured enthusiasm in terms of “work enthusiasm”, with no reference to any subject. In contrast, 16 [16] explicitly refer to an “enthusiasm for teaching the subject” (p. 3), and an instrument developed by Baier and colleagues [13] includes both generic and subject-related items. Clearly, the conceptualization and the dimensionality of enthusiasm for teaching are important for robust modeling. Thus, our first aim is to clarify if enthusiasm for teaching can be treated as a single dimension or if a generic and a subject-related dimensions should be considered separately.

#### Location of enthusiasm in the TPC and JD-R models

Enthusiasm for teaching is a facet of teacher enthusiasm, which is important for both teachers’ professional competence and health [5–7, 18, 19]. Of the four competence aspects described in the TPC model, enthusiasm is a key factor of *motivational orientations*, which are crucial for teachers’ psychological functioning [40]. Another aspect is *professional knowledge*, which includes pedagogical psychological knowledge (PPK) as a generic domain, and two subject-related domains: content knowledge (CK) and pedagogical content knowledge (PCK) [5, 41]. Third, teachers’ *beliefs*, *values*, *and goals* influence their attitudes towards students, the teaching profession and teaching quality. Finally, teachers’ *self-regulation* abilities are crucial for their management of personal resources and coping with job demands.

In the JD-R model, enthusiasm is an important work engagement indicator, together with for example strong identification with the profession [18]. This model is rooted in the assumption that every working individual faces job demands (e. g., challenging student behavior) that require physical and mental effort. Job resources (e. g., support from colleagues) and personal resources (e.g., self-confidence) can help to achieve professional goals, reduce burdens of job demands and promote professional development [18, 23, 30, 39]. Processes linked to job/personal resources and demands affect personal and organizational outcomes in contrasting ways. Strong resources lead through strong work engagement to desirable personal and organizational outcomes, as work engagement is positively related to well-being and performance [39]. Moreover, in contrast, mismatches between available resources and demands may result in emotional exhaustion and burnout, with costly consequences for employees’ health and organizations. For in-service teachers, such mismatch can cause major mental and physical health problems, with adverse effects on teaching quality, absenteeism, and career changes that can pose severe challenges for schools and governments [1, 2, 21, 42]. The same may hold for pre-service teachers, e.g. when qualified pre-service teachers decide to quit teacher education.

Accordingly, teacher enthusiasm is a component of both the TPC model, as an affective domain of teachers’ motivational orientations, and JD-R model, where it is located in the motivational path between personal/job resources and personal/organizational outcomes. Having described our conceptual frameworks, the following sections outline six cognitive and affective-motivational factors derived from these frameworks and recognized as important for enthusiasm for teaching: professional knowledge, academic self-concept, career choice motives, emotional exhaustion, intention to quit, and occupational commitment.

### Professional knowledge

Professional knowledge (CK, PCK, and PPK) is needed to meet the demands of the teaching profession, thus it plays a key role in the TPC model [5, 41, 43], governs the structure of teacher education at university [44], and is related to several desirable outcomes, like instructional quality [15] and students’ performance [45–48]. CK is teachers’ subject matter knowledge (including subject-related topics, concepts and contexts), PCK is the knowledge teachers need to make the content of a certain subject accessible to their students, and PPK is teachers’ knowledge of general teaching- and learning-related facets like classroom management and learning processes [5, 49, 50]. These domains of professional knowledge are predominantly acquired in the academic phase of teacher education [51]. Thus, since pre-service teachers are teacher *students*, the motivational component of enthusiasm for teaching could possibly lead to higher professional knowledge, which is required for teaching. This is in accordance with the JD-R model, in which enthusiasm potentially leads to higher performance [18].

### Academic self-concept

Self-concept refers to the evaluation of one’s own performance in certain domains, such as pre-service teachers’ *academic* performance. Academic self-concept is treated as a cognitive domain of motivational orientations in the TPC model. As an important characteristic of teachers, it promotes several desirable outcomes like teacher self-efficacy and teacher well-being [5, 52].

We assume that academic self-concept is relevant for enthusiasm of pre-service teachers because the perception of one’s own competence is an important prerequisite of intrinsic motivation [53], and thus most likely for enthusiasm. Moreover, self-concept and joy are positively correlated [54]. As joy and enthusiasm are conceptually closely related (e.g., [32]) a correlation between enthusiasm and self-concept seems plausible.

### Career choice motives

Career choice motives are the reasons for deciding to choose a particular profession. They are considered as intrinsic or extrinsic, whereby intrinsic motives for choosing teaching include personal interest in a specific subject and the desire to interact with children and adolescents, and extrinsic motives include the compatibility of work and family life, and expectations of parents and friends [53, 55, 56].

Career choice motives have clearly demonstrated links with development of professional competence [40, 57, 58] and burnout [59]. They warrant attention here because they influence pre-service teachers’ choice of profession and subsequent enthusiasm for teaching [60]. Moreover, *intrinsic* career choice motives are important motivational orientations [40]. In contrast, studies on teachers’ well-being indicate that burnout may be linked to *extrinsic* motives [59]. Thus, enthusiasm may be positively and negatively related to intrinsic and extrinsic career choice motives, respectively.

### Emotional exhaustion, the intention to quit, and occupational commitment

There are high risks for teachers leaving the teaching profession, even within a few years of starting [3, 28]. To prevent this, knowledge is needed of teachers’ motives for leaving the profession, which may include the frequent feeling of being overwhelmed by the high social and emotional demands of the job when they start [61, 62]. According to the JD-R model, if this experience continues and applied coping strategies fail to give relief, emotional exhaustion can occur, potentially leading to burnout and turnover intention [30, 31]. Teachers’ emotional exhaustion, a combination of emotional overload and lack of emotional resources [18, 63], is negatively related to both students’ and teachers’ performance [64, 65]. Enthusiasm has a mitigating impact on this process [24, 60], and given its importance we aimed to determine (1) if emotional exhaustion and enthusiasm are already related during teacher education at university, and (2) if pre-service teachers’ enthusiasm for teaching negatively predicts the intention to quit teacher education.

Teachers who leave the profession have less affective occupational commitment [66], i.e. emotional attachment to the profession, or *motivational orientations* towards it [61, 62]. Occupational commitment and enthusiasm for teaching are closely related as they are both facets of an affective orientation towards teaching-specific tasks. They mainly differ in degree of abstraction: enthusiasm for teaching is more specific and task-related than occupational commitment, which covers more global personal attitudes. Klassen and Chiu [62] found that occupational commitment decreases with in-service teachers’ years of experience. They also found that pre-service teachers had lower intention to quit than in-service teachers, but occupational commitment directly influenced both groups’ intention. They assumed that these differences occur because “novice teachers’ expectations of the work environment may be unrealistic, and must be recalibrated when the realities of day-to-day work intrude on the hoped-for learning environment” ([62], p. 122). Enthusiasm could, as shown for emotional exhaustion, counter these trends. Its potential relevance is supported by an important finding regarding the JD-R model that occupational commitment seems to be more strongly influenced by the motivational process than the health impairment process [20]. If so, the positive influences of job/personal resources and work engagement and enthusiasm on occupational commitment may outweigh negative influences of job demands and burnout. Thus, it is reasonable to foster enthusiasm for teaching of pre-service teachers. No previous studies have investigated the relationships between occupational commitment and teacher enthusiasm. However, these theoretical considerations indicate that pre-service teachers’ enthusiasm for teaching may have a positive relation with occupational commitment.

### Research questions and hypotheses

The study at hand aims to clarify the empirical structure of teacher enthusiasm and to identify cognitive and affective factors that may be related to pre-service teachers’ enthusiasm for teaching. The following research questions and hypotheses, based on recent research, guided the analysis:

1. Can pre-service teachers’ enthusiasm for teaching be parsimoniously treated as a one-dimensional construct, or should it be separated into generic (enthusiasm for teaching) and subject-specific (enthusiasm for teaching the subject) dimensions? We hypothesized that it can be considered as two-dimensional, similar to the dimensionality of professional knowledge and self-concept, where PCK- and PPK-related domains were shown to be separable [50, 67].

2. Is pre-service teachers’ enthusiasm for teaching related to the cognitive factors professional knowledge and academic self-concept? We assume, that enthusiasm is positive related with both the domains of professional knowledge and academic self-concept [51, 52].

3. How is pre-service teachers’ enthusiasm for teaching related to the following affective-motivational factors: career choice motives, emotional exhaustion, intention to quit, and occupational commitment? We hypothesized that: it is positively and negatively related to single and joint intrinsic and extrinsic career choice motives, respectively; negatively related to both emotional exhaustion and intention to quit; and positively related to occupational commitment [18, 62].

## Methodology and methods

### Sample and procedure

The study presented here was part of the longitudinal KeiLa (*Development of professional competence in pre-service mathematics and science teacher education*) investigation of individual and institutional determinants of pre-service mathematics and science teachers’ development of professional competence at 25 German universities [68]. Participants (*N* = 299) attended up to four 4-hour paper-and-pencil assessments in which they provided information on multiple aspects of professional competence of teachers including the ones of interest for this study. The participants received monetary compensation (10 € per hour). Since we were not interested in longitudinal analyses, we chose data from each participants’ first attendance, gaining a cross-sectional sample. Teaching experience was used as a filter variable as teaching experience is crucial for answering our research question. Thus, only pre-service teachers who had some teaching experience were included, leading to a sample of 211 pre-service teachers from 20 universities. Of the participants, 163 were female (77.3%) and 48 were male (22.7%). Their mean age at first attendance was 21.54 years (*SD* = 2.59). Participants were enrolled in semesters 1 to 9 with n_1_ = 79 (37.4%), n_3_ = 25 (11.8%), n_5_ = 81 (38.4%), n_7_ = 24 (11.4%), and n_9_ = 2 (1%).

### Measures

Wherever mentioned, the 4-point Likert scale is 4 = fully applies; 3 = largely applies; 2 = does not much apply; 1 = does not apply at all; α refers to Cronbach’s alpha.

#### Enthusiasm for teaching

We assessed enthusiasm for teaching with 12 4-point Likert-type items of an instrument including two subscales, each with six items, designed to probe: generic enthusiasm for teaching (*M* = 21.61, *SD* = 2.24, α = .82), and enthusiasm for teaching the subject (*M* = 21.65, *SD* = 2.23, α = .82; see S1 File for the complete instrument). The instrument was developed for the KiL project (*Measurement of professional competences in mathematics and science teacher education*, e.g., [69]).

#### Cognitive determinants

We regarded professional knowledge and academic self-concept as possible cognitive determinants of pre-service teachers’ enthusiasm for teaching. Thus, we measured our participants’ professional knowledge in the CK, PCK, and PPK domains with a knowledge test, using items developed in the KiL project. We calculated Weighted Likelihood Estimation (WLE) scores [70] for each domain using the R package ‘TAM’ [71]. To capture their CK and PCK we used 34 items related to ecology, genetics, evolution, morphology, and physiology, and 38 items regarding instructional strategies and students’ understanding, respectively (CK_WLE_(Rel) = .74, PCK_WLE_(Rel) = .62) [72, 73]. To capture PPK, we applied four subscales covering (1) teaching (TE), (2) learning and development (LD), (3) performance assessment (PA), and (4) classroom management (CM), with 29, 34, 19 and 25 items, respectively [49]. These yielded acceptable to good WLE reliabilities of TE_WLE_(Rel) = .77, LD_WLE_(Rel) = .78, PA_WLE_(Rel) = .60, CM_WLE_(Rel) = .80, respectively.

To measure academic self-concept we adapted an instrument developed by Retelsdorf and colleagues [74]. This included five 4-point Likert type items designed to probe each of the three dimensions, related to (1) CK, (2) PCK and (3) PPK ((1) *M* = 15.71, *SD* = 2.6, (2) *M* = 14.92, *SD* = 2.54, and (3) *M* = 14.41, *SD* = 2.89 with α = .85, .84, and .87, respectively).

#### Affective-motivational determinants

We assumed that affective-motivational factors related to pre-service teachers’ enthusiasm for teaching include career choice motives, emotional exhaustion, occupational commitment, and intention to quit the teacher education program.

Career choice motives were assessed using an instrument developed by Pohlmann and Möller [55]. Three subscales (measured by 4-point Likert type items) cover three intrinsic motives: (1) pedagogical interest, (2) interest in a particular subject, and (3) teaching ability beliefs (6, 4 and 5 items; (1) *M* = 21.39, *SD* = 2.81, (2) *M* = 14.25, *SD* = 1.82 and (3) *M* = 16.86, *SD* = 2.29; α = .86, .76, and .78, respectively). Another three subscales cover the extrinsic motives (1) utility beliefs, (2) low difficulty of teacher education, and (3) social influences (8, 4, and 5 items; (1) *M* = 22.48, *SD* = 5.34, (2) *M* = 6.28, *SD* = 2.35, and (3) *M* = 11.15, *SD* = 3.5; α = .89, .85, and .76, respectively).

To capture emotional exhaustion we applied an instrument originating from the *Maslach Burnout Inventory* (MBI; [75]) adapted for PISA/COACTIV studies [76]. This consists of four 4-point Likert type items, which yielded scores of *M* = 8.90, *SD* = 2.33, with α = .74.

To obtain information about intention to quit the teacher education program, we used five 5-point Likert type items (5 = fully applies, 4 = largely applies, 3 = uncertain, 2 = does not much apply, 1 = does not apply at all; *M* = 7.20, *SD* = 2.46; α = .65). The items were developed for the KiL-project.

To assess occupational commitment, a subscale of the *Occupational Commitment* instrument, originally developed to examine nurses [66], was adapted for teaching. This includes six 4-point Likert type items (*M* = 21.13, *SD* = 2.37, α = .67).

### Data analysis

#### Dimensionality of enthusiasm for teaching

The operationalization of enthusiasm for teaching is vague [13, 24, 40]. To obtain deep understanding of the importance of academic teacher education for enthusiasm, knowledge of its empirical structure is needed (research question 1). We applied confirmatory factor analysis (CFA) using Mplus software [77] to assess whether enthusiasm for teaching can be parsimoniously treated as a single dimension (Model 1) or should be divided into generic and subject-related dimensions (Model 2). As the items were answered on an ordered categorical Likert-scale, we chose a robust Weighted Least Squares Mean and Variance Adjusted estimator. To assess the significance of differences in the models’ fit we used the χ^2^-based DIFFTEST option in Mplus [77].

#### Cognitive and affective-motivational factors interacting with teaching enthusiasm

To test our hypotheses regarding research question 2, that enthusiasm for teaching is related to professional knowledge and academic self-concept, we specified enthusiasm for teaching as a latent variable composed of the applied items. To assess the relationships between pre-service teachers’ enthusiasm for teaching and the domains of professional knowledge, we calculated MIMIC (*Multiple Indicators Multiple Cause*) regression modeling implemented in Mplus [77] with enthusiasm as a latent independent variable. We assessed relationships between enthusiasm for teaching and dimensions of academic self-concept by bivariate correlation modeling using Mplus [77].

To evaluate relationships between enthusiasm for teaching and affective-motivational variables (research question 3) we first specified MIMIC models to assess its relations with career choice motives with enthusiasm as the latent dependent variable. In detail, we specified three models for enthusiasm and the intrinsic career choice motives subject-specific interest (Model 1), pedagogical interest (Model 2), and ability beliefs (Model 3), respectively, and three models for enthusiasm and the extrinsic career choice motives utility beliefs (Model 5), low difficulty (Model 6), and social influences (Model 7), respectively. Additionally, we also assessed relations between enthusiasm for teaching and joint intrinsic (Model 4) as well as joint extrinsic motives (Model 8) in additional multiple regression analyses as career choice motives can further be modeled with joint intrinsic and joint extrinsic motives as secondary factors [55]. Second, as we assume a reciprocal relationship between enthusiasm for teaching and emotional exhaustion, we applied correlation analyses [18, 31]. Third, MIMIC models with enthusiasm as the latent independent variable were modeled to gain information about the relationship of enthusiasm for teaching and intention to quit as well as occupational commitment.

### Ethics statement

All participants participated voluntarily and gave their consent for inclusion prior to every assessment. The purpose of the study (longitudinal assessment of individual and institutional determinants of pre-service teachers’ development of professional competence) was explained in advance. Payment information was collected to pay participants their compensation. This information has been linked at no time to the other data of the study. The study was conducted in accordance with the Declaration of Helsinki. As data collection was anonymously proceeded in the familiar surroundings of university lecture halls, therefore causing no distress to the participating pre-service teachers, no ethical approval of the local Ethics Committee was necessary.

## Results

### Dimensionality of enthusiasm for teaching

Our CFA, which we applied to investigate the empirical structure of enthusiasm for teaching (research question 1), suggested that Model 1 and Model 2 fitted the data similarly (according to CFI, TLI, and RMSEA values; Table 1). Accordingly, the χ^2^-difference test detected no significant difference between their fits: χ^2^(1) = 0.274, *p* = 0.601. Thus, the more parsimonious Model 1 was retained, as its restrictions cannot be rejected, and enthusiasm for teaching was treated as one-dimensional in further analyses.

**Table 1:** Goodness-of-fit indices of the two models of enthusiasm for teaching.

Model	χ^2^	*df*	RMSEA	CFI	TLI
**Model 1: one-factor**	465.22	54	0.19	0.93	0.92
**Model 2: two-factor**	495.45	53	0.20	0.93	0.91

χ2 = Chi-square, *df* = Degrees of freedom, RMSEA = Root mean square error of approximation, CFI = Comparative fit index, TLI = Tucker-Lewis index.

### Cognitive variables

#### Professional knowledge

Contrary to our hypotheses, the regression analysis revealed no significant relationships between enthusiasm for teaching and the considered dimensions of professional knowledge (Table 2).

**Table 2:** Results of the regression analysis for the relationships between indicated dimensions of professional knowledge and enthusiasm for teaching.

Parameter	β	*SE*	*R^2^*
**CK**	.01	0.07	.00
**PCK**	.00	0.07	.00
*PPK*			
** Teaching**	.10	0.07	.01
** Learning and development**	.07	0.09	.00
** Performance assessment**	.02	0.07	.00
** Classroom management**	.04	0.07	.00

β = Standardized regression coefficient, *SE* = Standard error, *R^2^* = Coefficient of determination.

#### Academic self-concept

To investigate the relationships between enthusiasm for teaching and the three dimensions of academic self-concept we calculated bivariate correlations (Table 3). As we hypothesized, results revealed significant but small positive relationships between enthusiasm for teaching and CK-, PCK- and PPK-related academic self-concept.

**Table 3:** Results of the correlation analysis for relationships between enthusiasm for teaching and indicated dimensions of academic self-concept.

Parameter	*r*	*SE*
**Self-concept CK**	.07*	0.03
**Self-concept PCK**	.13***	0.03
**Self-concept PPK**	.12***	0.03

*r* = Unstandardized regression coefficient, *SE* = Standard error.

*p < .05

***p < .001.

### Affective-motivational determinants

#### Career choice motives

Three separate MIMIC models (1–3) revealed significant relationships between enthusiasm for teaching and the dimensions of intrinsic career choice motives: subject-specific interest, pedagogical interest, and ability beliefs. A combined model (Model 4) confirmed the relationships between enthusiasm for teaching and both pedagogical interest and ability beliefs. In contrast, we found no significant relationship between enthusiasm for teaching and the extrinsic career choice motives utility beliefs, low difficulty, or social influences, either separately considered in linear regression models (Models 5–7) or jointly considered in a multiple regression model (Model 8). See Table 4 for an overview.

**Table 4:** Results of the regression analysis for the relationships between enthusiasm for teaching and indicated career choice motives (standardized regression coefficients; standard errors in parenthesis).

Parameter	Model 1	Model 2	Model 3	Model 4	Model 5	Model 6	Model 7	Model 8
*Intrinsic career choice motives*								
**Subject-specific interest**	.25*** (.07)			.07 (.06)				
**Pedagogical interest**		.48*** (.06)		.40*** (.06)				
**Ability beliefs**			.39*** (.06)	.25*** (.06)				
*Extrinsic career choice motives*								
**Utility beliefs**					-.08 (.07)			-.07 (.09)
**Low difficulty**						-.11 (.07)		-.15 (.11)
**Social influences**							-.10 (.08)	.15 (.1)
***R*** ^***2***^	.06	.23	.16	.31	.01	.01	.01	.03

***p < .001.

#### Emotional exhaustion

Correlation analysis revealed a small significantly negative relationship indicating that enthusiasm for teaching decreases with increasing emotional exhaustion (*r* = -.07, *SE* = 0.03, *p* < 0.05).

#### Intention to quit the teacher education program and occupational commitment

The remaining two regression analyses with occupational commitment and intention to quit the teacher education program regressed on enthusiasm for teaching as a latent independent variable, respectively, reveal a significantly positive relationship of enthusiasm for teaching with occupational commitment, and a significantly negative relationship with intention to quit (see Table 5).

**Table 5:** Results of the regression analysis for the relationships between intention to quit and occupational commitment with enthusiasm for teaching.

Scale	β	*SE*	*R^2^*
**Intention to quit**	-.21***	0.05	.10
**Occupational commitment**	.27***	0.03	.28

β = Standardized regression coefficient, *SE* = Standard error, *R^2^* = Coefficient of determination.

***p < .001.

## Discussion

Our approach to gain deeper insights into the conceptualization and the structure as well as the relations of pre-service teachers’ enthusiasm for teaching with the help of the TPC and JR-D models yielded to interesting findings, as summarized and discussed below.

Teachers are experts in their subject, teaching in general, and teaching the subject, which are reflected in the structures of both professional knowledge [78] and academic-self-concept [50, 79]. Accordingly, with regard to our first research question, we hypothesized that enthusiasm for teaching should be modelled as a two-dimensional (generic and subject-related) construct, but our results suggest that treating it more parsimoniously as a one-dimensional construct does not compromise explanatory power. This may have been due to our participants having too little teaching experience to clearly differentiate between generic and subject-related aspects in their conceptualization of teaching. Thus, they may not have been sufficiently aware of the object of their enthusiasm (teaching in general vs. teaching the subject).

To answer our second research question, we explored the relationship of enthusiasm for teaching and the cognitive variables professional knowledge and academic self-concept. In contrast to a positive relationship between enthusiasm for teaching and academic self-concept, we found no indication that enthusiasm for teaching is related to professional knowledge. The domains of professional knowledge are foundations of teacher education, and professional knowledge is eventually needed for successful teaching [80]. Enthusiasm for teaching being not a predictor for pre-service teachers’ performance may be explained by its target, which is teaching (a subject), while professional knowledge is a prerequisite for teaching in the first place [81]. Thus, pre-service teachers are possibly not aware of the important relationship between their knowledge and teaching. However, the *experience* of one’s own competence and performance is an important aspect of intrinsic motivation [53]. Accordingly, we found a correlation of self-concept with enthusiasm for teaching.

With our third research question we aimed to explore the relationship of enthusiasm for teaching and multiple affective-motivational determinants. First, regarding the joint model of the three intrinsic career choice motives and enthusiasm (Model 4), we found significant positive relationships between enthusiasm for teaching and two intrinsic career choice motives–pedagogical interest and ability beliefs–, as expected. In contrast, although predominance of external motives is reportedly linked to burnout [29], we did not detect a negative relationship between external motives and enthusiasm for teaching, neither in the respective single models (Models 5–7) nor in the joint model of all three extrinsic career choice motives (Model 8). However, findings from person-centered studies on career choice motives indicate that teachers have both intrinsic and extrinsic reasons for engaging in teaching [82, 83]. Hence, a more holistic approach, recognizing fluctuating contributions of both internal and external career choice motives may be required. Second, in accordance with findings for in-service teachers [18, 60], we detected the hypothesized negative correlation between pre-service teachers’ emotional exhaustion and enthusiasm for teaching. Hence, high enthusiasm for teaching seems to diminish emotional exhaustion that occurs during teacher education at university, and vice versa, high emotional exhaustion during teacher education has an unfavorable influence on enthusiasm for teaching. This clearly highlights the importance of enthusiasm for teaching in this early stage of the teaching career. Third, as further assumed, we found that enthusiasm for teaching was positively and negatively associated with occupational commitment and the intention to quit teacher education, respectively. This corroborates the importance of enthusiasm for teaching in pre-service stages. However, as Klassen and Chiu [62] found that occupational commitment decreases with in-service teachers’ years of experience, it is also important to identify ways to maintain high enthusiasm and thus commitment.

### Limitations

As our sample was cross-sectional, we cannot draw conclusions about the direction of detected effects or changes in measured variables with time. However, we identified factors that apparently relate to pre-service teachers’ enthusiasm for teaching during teacher education at university, and our results provide indications of potentially fruitful foci of future research. These include subject-specific enthusiasm, especially aspects associated with subject-related career choice motives and self-concept, which we did not explicitly address. As mentioned above, a possible explanation of the apparent uni-dimensionality of enthusiasm for teaching is that our pre-service teachers did not think about teaching in isolation from the subject. This may have been due to priming, as enthusiasm for teaching was tested in the framework of a larger study, and most items were related to their respective subjects (e.g., biology). Overall, since all associations we found were of self-reported constructs, we have to consider common method bias effects [84], which indicate that explained variance may be influenced by sharing the same assessment method. While professional knowledge was assessed with a knowledge test, all other measurements were based on self-reports. Further studies should take this into account and variate or combine methods for assessing enthusiasm for teaching and related constructs.

## Implications

### Implications for future research

Combined use of the TPC and JD-R models enabled identification of important factors for pre-service teachers’ enthusiasm for teaching. This highlights its importance for both professional competence *and* health, as well as the value of combining multiple frameworks for a holistic investigation of enthusiasm for teaching. Clearly, promoting both teachers’ competence and health is important, so health-related aspects should be more frequently addressed when teachers’ professionalization is considered, and could clarify pre-service teachers’ requirements for a successful start in the profession.

When investigating enthusiasm for teaching, more attention should be paid to its conceptualization and operationalization. As mentioned, we suspect that our participants’ responses were influenced by a strong link between teaching and the subject. Thus, the possibility that instruments or procedures used to probe it include explicit or implicit references to subjects should be considered.

An interesting previous finding is that career choice motives can change during a professional career. Thus, teachers’ reasons for choosing and remaining in the profession depend on their individual experiences over time [60, 85]. Problems like burnout may occur if teachers’ enthusiasm diminishes for any reasons, and extrinsic motives prevail [59, 82]. Longitudinal analyses would help to illuminate such temporal processes and associated factors.

### Implications for teacher education

As enthusiasm is important for both professional competence and health, validated results from studies like ours should clearly be applied in teacher education. Our participants apparently had remarkably high enthusiasm for teaching (*M* = 43.27, *SD* = 4.15; max. of 48). It would be very desirable to maintain high enthusiasm during their professional career, and to identify reasons for any declines, as enthusiasm for teaching is also positively related to occupational commitment and negatively affecting the intention to quit teacher education, and hence the profession. However, the working conditions at school often differ from pre-service teachers’ expectations [86]. Some can only meet the various demands of teaching with great emotional and personal effort because they have not learnt to apply appropriate coping strategies [87]. The risk for emotional exhaustion and burnout is high for these teachers, but enthusiasm can help to meet these demands already in the first phase of teacher education, as is shown in this study. Thus, it is important to identify ways to foster enthusiasm for teaching during teacher education at university, and consideration of influential factors we identified may be helpful.

Academic self-concept as well as intrinsic career choice motives are positively related to enthusiasm for teaching, indicating that pre-service teachers with a positive academic self-concept, and particularly intrinsic career choice motives, are more enthusiastic. Often, teacher education does not explicitly address self-concept or career choice motives, despite their relevance. Hence, teacher education courses should be adjusted to promote pre-service teachers’ frequent reflection on their motives for choosing the teaching profession. They should know that there are intrinsic and extrinsic motives, which have differing relations to their enthusiasm. Moreover, pre-service teachers should be motivated to reflect on their perceptions of their own performance and their emotions, for example with courses to improve knowledge of emotions and its regulation as designed by Carstensen and colleagues [88], which can significantly improve the well-being and health of pre- and in-service teachers [24, 88].

Finally, pre-service teachers should learn that good teachers require not only competence but also health, and the importance of enthusiasm could be potentially clarified using the TPC and JD-R models in teacher education.

## Supporting information

S1 File Instrument enthusiasm for teaching. (DOCX)

S1 Dataset Contains data of scales enthusiasm for teaching, intention to quit teacher education, academic self-concept, career choice motives, emotional exhaustion, occupational commitment, pedagogical content knowledge, and content knowledge. (SAV)
