# Supplementary material for: Examining the dimensionality of pre-service teachers’ enthusiasm for teaching by combining frameworks of educational science and organizational psychology
Source: PLoS One. 2021 Nov 18;16(11):e0259888. doi: 10.1371/journal.pone.0259888 (PMC8601546; doi:10.1371/journal.pone.0259888)
Supplement: S1 File — (DOCX) [file pone.0259888.s001.docx]

**Instrument: Enthusiasm for teaching**

1. I teach with great enthusiasm.

The subject of biology

In general

1. I really enjoy teaching.

The subject of biology

In general

1. Teaching is one of my favorite activities.

The subject of biology

In general

1. I always enjoy teaching students new things.

The subject of biology

In general

1. Interacting with students is one of the nicest aspects of the teaching profession for me.

The subject of biology

In general

1. It’s a pleasure to teach.

The subject of biology

In general
